# Cerebrospinal fluid micro-volume changes inside the spinal space affect intracranial pressure in different body positions of animals and phantom

**DOI:** 10.3389/fnmol.2022.931091

**Published:** 2022-09-14

**Authors:** Marijan Klarica, Milan Radoš, Gorislav Erceg, Ivana Jurjević, Antonio Petošić, Zdravko Virag, Darko Orešković

**Affiliations:** ^1^Department of Pharmacology and Croatian Institute for Brain Research, School of Medicine, University of Zagreb, Zagreb, Croatia; ^2^Department of Electroacoustics, Faculty of Electrical Engineering and Computing University of Zagreb, Zagreb, Croatia; ^3^Department of Fluid Mechanics, Faculty of Mechanical Engineering and Naval Architecture, University of Zagreb, Zagreb, Croatia; ^4^Department of Molecular Biology, Ruder Bošković Institute, Zagreb, Croatia

**Keywords:** CSF pressure, CSF volume changes, body position, phantom, subatmospheric CSF pressure

## Abstract

Interpersonal differences can be observed in the human cerebrospinal fluid pressure (CSFP) in the cranium in an upright body position, varying from positive to subatmospheric values. So far, these changes have been explained by the Monroe–Kellie doctrine according to which CSFP should increase or decrease if a change in at least one of the three intracranial volumes (brain, blood, and CSF) occurs. According to our hypothesis, changes in intracranial CSFP can occur without a change in the volume of intracranial fluids. To test this hypothesis, we alternately added and removed 100 or 200 μl of fluid from the spinal CSF space of four anesthetized cats and from a phantom which, by its dimensions and biophysical characteristics, imitates the cat cerebrospinal system, subsequently comparing CSFP changes in the cranium and spinal space in both horizontal and vertical positions. The phantom was made from a rigid “cranial” part with unchangeable volume, while the “spinal” part was made of elastic material whose modulus of elasticity was in the same order of magnitude as those of spinal dura. When a fluid volume (CSF or artificial CSF) was removed from the spinal space, both lumbar and cranial CSFP pressures decreased by 2.0–2.5 cm H_2_O for every extracted 100 μL. On the other hand, adding fluid volume to spinal space causes an increase in both lumbar and cranial CSFP pressures of 2.6–3.0 cm H_2_O for every added 100 μL. Results observed in cats and phantoms did not differ significantly. The presented results on cats and a phantom suggest that changes in the spinal CSF volume significantly affect the intracranial CSFP, but regardless of whether we added or removed the CSF volume, the hydrostatic pressure difference between the measuring sites (lateral ventricle and lumbar subarachnoid space) was always constant. These results suggest that intracranial CSFP can be increased or decreased without significant changes in the volume of intracranial fluids and that intracranial CSFP changes in accordance with the law of fluid mechanics.

## Introduction

It is known that changing the body's position from horizontal to vertical leads to significant changes in the cerebrospinal fluid pressure (CSFP) within the cranium, which varies from positive to negative (subatmospheric) values (Davson et al., 1987; Andresen et al., 2015; Farahmand et al., 2015). This phenomenon is explained by the Monroe–Kellie doctrine and the classical hypothesis of –cerebrospinal fluid physiology. According to the Monroe–Kellie doctrine, intracranial CSFP depends on the interaction of three volumes that fill the cranium: the volumes of blood, brain, and cerebrospinal fluid. After changing the body's position from horizontal to vertical, it is generally accepted that there is a redistribution of venous blood from the cranium to the lower parts of the body, accompanied by the collapse of internal jugular veins (Qvarlander et al., 2013; Holmlund et al., 2018). Inside the cranial space, partial collapse of venous vessels (observed during the cranial opening in neurosurgical operations) might reduce intracranial CSFP (Davson et al., 1987). In addition, it is considered that there is a rapid and short-term movement of part of the intracranial volume of cerebrospinal fluid into the spinal subarachnoid space (Magnaes, 1976a,b; Magnaes, 1989; Alperin et al., 2005a,b). Thus, CSFP decreases due to the simultaneous reduction of the two volumes filling the cranium (blood and CSF), but this change in intracranial CSFP is considered transient. A well-known Davson's equation (Intracranial CSFP = Vf x Ro + Pv, where Vf = rate of secretion; Ro = the resistance to the flow (circulation) of CSF along the CSF system; Pv = the resistance to absorption of the CSF into the venous sinuses/blood circulation) connects CSFP value with the classical concept of CSF secretion, unidirectional circulation, and absorption (Davson et al., 1987). Thus, according to the classical hypothesis of cerebrospinal fluid physiology, due to the constant formation of cerebrospinal fluid within the cerebral ventricles, the decrease in cerebrospinal fluid volume after changing the body's position from horizontal to vertical would be quickly compensated, and intracranial CSFP would take on positive values (Magnaes, 1976a,b; Marmarou et al., 1978; Davson et al., 1987).

In our previous research on cats and phantom (whose biophysical and anatomical characteristics mimic the CSF system of cats), it was observed that CSFP in the vertical position depends on the measuring point, and it varies from about−4 cm H_2_O (subatmospheric) in the lateral ventricle (LV) to about +32 cm H_2_O in the lumbar subarachnoid space (LSS) (Klarica et al., 2014; Orešković et al., 2017b). However, the negative CSFP value in LV was stable, as well as the hydrostatic gradient value between LV and LSS, while the animal was in the head-up position (Klarica et al., 2014). Furthermore, the measurement of CSFP from animals did not differ from those observed on the phantom, in which there was no change in volume in the “cranial” part when changing position. These findings have led to the development of a new hypothesis that CSFP changes in accordance with the law of fluid mechanics and that the reduction of intracranial CSFP occurs without visible changes in intracranial fluid volume (Klarica et al., 2006, 2014; Orešković et al., 2017a,b, 2018). This hypothesis is further supported by new observations in humans that there are almost no changes in intracranial vessels when changing body position from supine to sitting position (Kosugi et al., 2020).

In the upright position, there is a large interindividual difference in intracranial CSFP values (Magnaes, 1976a,b; Davson et al., 1987; Chapman et al., 1990). As the cerebrospinal fluid volume does not change significantly in the upright position in the cranium, one of the reasons for this CSFP variability could be the existence of a different cerebrospinal fluid volume in the spinal compartment.

We intended to examine whether changes in CSF volume inside the lumbar subarachnoid space (LSS) would lead to changes in intracranial CSFP without significant changes in CSF volume inside the cranium. For this purpose, the same fluid volume will be added or removed from the LSS of cats and the phantom (see Material and methods section), and CSFP will be measured inside the LV and the LSS in horizontal and vertical positions. We expect that the results of this study will demonstrate the dominant role of spinal CSF space in the regulation of intracranial CSFP and compliance of craniospinal space, which is essential for the understanding of the variety of physiological conditions and CSF-related neurological diseases.

## Materials and methods

### Animal experiments

The study was performed on male and female adult cats (2.2–3.4 kg body weight). The animals were kept in cages with natural light-dark cycles and free access to water and food (SP215 Feline, Hill's Pet Nutrition Inc., Topeka, KS, USA).

### Ethics statement

**Figure 1:**
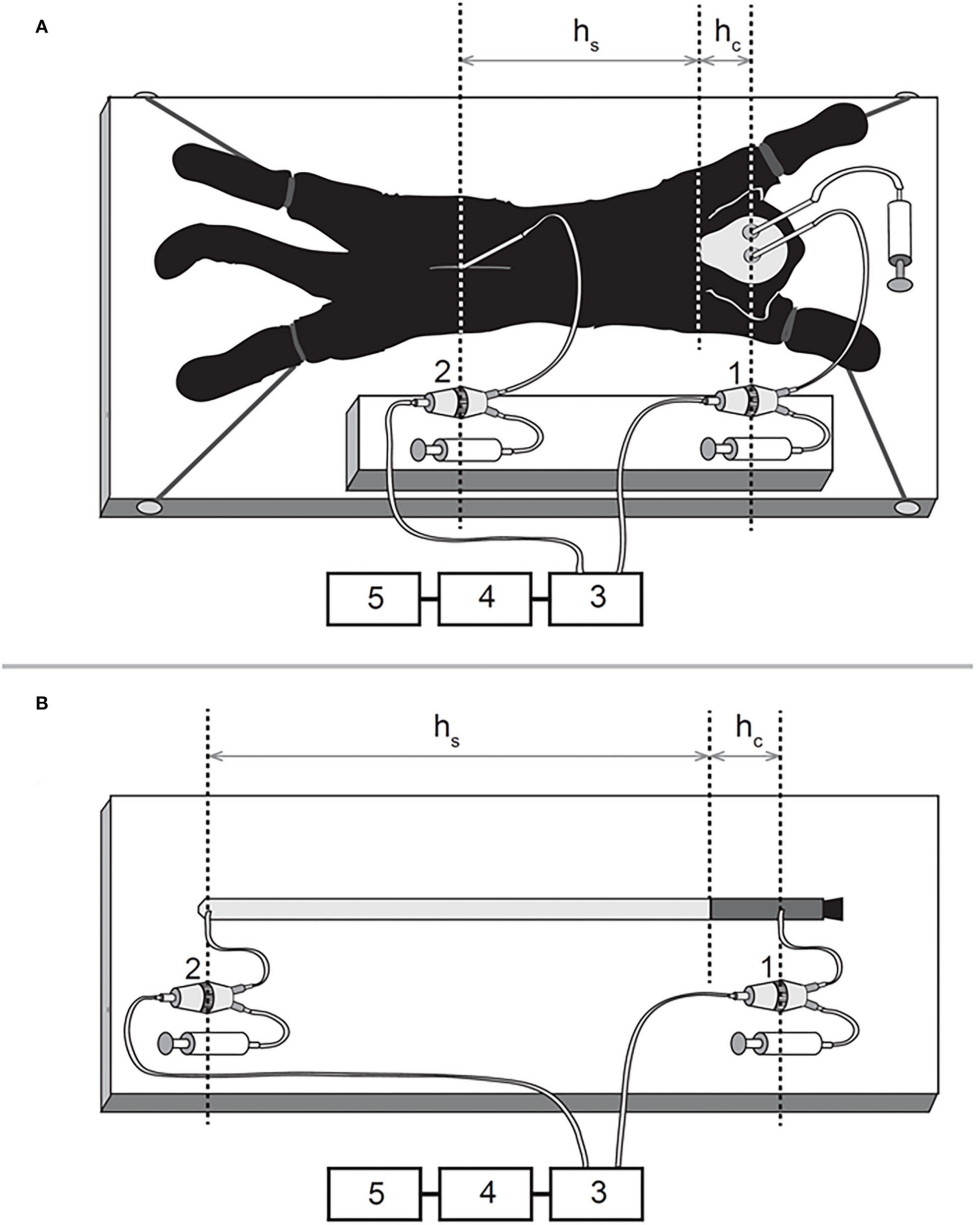
Schematic representations of the experimental models on a cat **(A)** and phantom **(B)** in horizontal position 1, pressure transducer connected to the cannula inside the lateral ventricle; 2, pressure transducer connected to the cannula inside the lumbar subarachnoid space; 3, Quand Bridge; 4, PowerLab/800; 5, personal computer; hc, the distance between the cisterna magna and the pressure measuring point inside the lateral ventricle; hs, the distance between the cisterna magna and the pressure measuring point inside the lumbar subarachnoid space.

The animals were in quarantine for 30 days, and the experiments were performed in accordance with the Croatian Animal Welfare Act. The protocol was approved by the Ethics Committee of the University of Zagreb Medical School (Approval No. 04-76/2006-18). Experiments shown in the manuscript were performed more than 15 years ago. At that time, the Croatian Animal Welfare Act allowed us to obtain experimental animals from private owners (domestic breeding). However, today in Croatia, we have a new Animal Welfare Act by which it is possible to obtain experimental animals only from official suppliers. The owners were verbally informed about the experimental protocol, which was previously approved by the official ethical committee. All efforts were made to minimize animal suffering, and all surgery, according to the protocol, was performed under anesthesia. The cats were anesthetized with α-chloralose (Fluka; 100 mg/kg i.p.) and fixed in a stereotaxic head holder (David Kopf, Tununga, CA, USA) in the sphinx position. The femoral artery was cannulated, the blood pressure was recorded *via* a T-connector, and blood samples were taken for analysis of the blood gases. No significant changes, either in blood pressure or blood gases, were observed during these experiments on cats, which continued breathing spontaneously under the α-chloralose anesthesia. A stainless steel cannula (0.9 mm ID) was introduced into the left LV at 2 mm lateral and 15 mm anterior to the stereotaxic zero point and 10–12 mm below the dural surface. A second cannula was placed in the right LV at the same position as the cannula in the left LV (Laitinen, 1968). The cannula in the right LV was used to measure intracranial CSFP. In order to measure the spinal CSFP in the lumbar region, a laminectomy (5 x 10 mm) of the L3 vertebra was performed. After the spinal dura and arachnoid incision, a third plastic cannula (0.9 mm ID) was introduced into the subarachnoid space. Leakage of CSF was prevented by applying cyanoacrylate glue to the dura around the cannula. Bone openings in the cranium and vertebra were hermetically closed by the application of a dental acrylate. After setting the measuring cannulas, the cat was removed from the stereotaxic device and then fixed in a prone position on a board (Figure 1A). CSF pressures were recorded using pressure transducers (Gould P23 ID, Gould Instruments, Cleveland, OH, USA) which were connected to a system that transformed analogous to digital data (Quand Bridge and PowerLab/800, ADInstruments, Castle Hill, NSW, Australia), and then entered into a computer (IBM, White Plains, NY, USA) (Figure 1A). Pressure transducers were calibrated using a water column, and the interaural line was taken as zero pressure. Instruments for pressure measurement were fixed on the board in such a way that the membrane of each transducer was at the same hydrostatic level as the corresponding measuring cannula, so there was no need to additionally adjust the transducers during the body position changes (Figure 1A) (Klarica et al., 2014). CSFP changes were recorded at 15-min intervals in horizontal (0°) and head-up (vertical; 90°) positions (Figure 1A).

### Experiments on a phantom

A new phantom model of the CSF system is made of two different materials which represent the main biophysical characteristics of the cranial (unchangeable volume) and spinal (changeable volume) parts of the CSF system (Figure 1B) (Klarica et al., 2014). In the construction of the phantom “CSF system,” we took into account the anatomical dimensions of the CSF system in cats. The “cranial” part is made of a plastic tube, 6 cm long, with an inner diameter of 0.6 cm and a wall thickness of 2.0 mm (Figures 1, **4**). This length of the plastic tube with a rigid wall is chosen because it represents the mean distance from the frontal sinuses to the foramen magnum, as found in 5 cats on x-rays of the animals' skulls (Klarica et al., 2014). The “spinal” part is made of a rubber balloon that is 31 cm in length (Natural Rubber Latex, Gemar, Casalvieri, Italy). This length is similar to the mean distance between the cisterna magna and the LSS at the level L3 vertebra where the pressure in cats was measured. The measuring cannula in the “cranial” part of the phantom was placed 4 cm proximally from the lower end of the plastic tube (Figure 1B), which corresponded to the distance between the cranial cannula in LV and the foramen magnum in cats. The second cannula was placed at the base of the rubber balloon so that the total distance between the two measuring cannulas was 35 cm (Figure 1B). Before measuring the pressures, the phantom was filled with artificial CSF without the presence of air bubbles and fixed on the board. The pressure transducers (Gould P23 ID, Gould Instruments, Cleveland, OH, USA) were fixed at the same level as the measuring cannulas and connected to the computer *via* an amplifier (QUAD Bridge and PowerLab/800, ADInstruments Ltd., Castle Hill, NSW, Australia) (Figure 1B). The pressures were measured in the same positions as in the cats (horizontal and vertical positions). The rubber balloon used to create the “spinal” part of the phantom had two modules of elasticity (Jurjević et al., 2011). Those modules were of the same order of magnitude as dural elasticity modules in big experimental animals (Tunturi, 1977; Kenning et al., 1981; Rosner and Coley, 1986). It was possible to stretch that balloon, especially in the horizontal plane, similar to the animal dura mater. Namely, in the craniocaudal direction, the dura mater is almost maximally stretched, while the stretching in the horizontal direction is possible because of the arrangement of collagen fibers (Kenning et al., 1981; Rosner and Coley, 1986). “CSFP” changes were recorded in phantom at 15-min intervals in horizontal (0°) and vertical (90°) positions (Figure 1B).

### CSFP measurement in experimental animals during lumbar CSF volume changes

In this experimental series, we aimed to verify if an increase or decrease of CSF volume inside the lumbar part of the CSF system leads to an increase or decrease in CSFP inside the cranial part. First, initial pressure values (control) were measured in cats fixed on a measuring board in a horizontal position (0°; Figure 1A). After the initial evaluation, 100 μL of artificial CSF was added to the LSS *via* T-connector, and pressure values were recorded in both the lumbar and cranial parts of the CSF system. When pressure values dropped to initial values, 200 μL of artificial CSF was added into the lumbar space, and the measurement was repeated. After returning to control values, 100 μL of CSF was extracted from the lumbar part and the pressure was measured again. When pressure returned to control values, a volume of 200 μL of CSF was extracted from the same space, and pressure measurement was repeated. The described measurements were then carried out in a head-up (vertical) position (90°; Figure 1A) in the same volume extraction and addition order.

### “CSFP” measurement in phantom during “lumbar” CSF volume changes

The experiments on the phantom were done in the same way. Through the cannula positioned in the “lumbar” part of the phantom in a horizontal position (0°; Figure 1B), the volume of artificial CSF was increased or decreased in the “lumbar” space, and CSFP was measured in the “cranial” and the “spinal“ parts of the phantom “CSF system.” After the measurement of control values, 100 μL of artificial CSF is added into the “lumbar space” *via* T-connector, and the pressure values are recorded in both the “lumbar” and “cranial” parts of the “CSF system.” When the pressure returns to control values by taking out the experimentally added volume of artificial CSF (100 μL), 200 μL of artificial CSF is added into the “lumbar” space, and measurement is repeated. After returning to control values by taking out the experimentally added volume of artificial CSF (200 μL), 100 μL of CSF is extracted from the “lumbar” part, and pressure is measured again. When pressure values return to normal by adding the experimentally removed volume of artificial CSF (100 μL), a volume of 200 μL of CSF is extracted from the same space, and pressure measurement is repeated. The described measurements are then carried out in a “head-up” (vertical) position (90°; Figure 1B) in the same volume extraction and addition order.

### Compliance within craniospinal space

Compliance is a ratio between the changes in CSF volume and CSF pressure expressed in mL/cm H_2_O. It is calculated in animals and phantom after the addition/ removal of a fluid volume from the spinal part of the system. The calculation has been done for both horizontal and vertical positions.

### Statistical analysis

Data are shown as a mean value ± standard error of the mean (SEM). A statistical analysis of all of the results was performed using the paired Student's *t*-test and ANOVA for repeated measures, with “condition” (cranial part and lumbar part) and position (0° and 90°) as a within-subject variable. In addition, a 2 x 2 mixed ANOVA was conducted on CSFP in a head-up position (90°), with “condition” (cranial part and lumbar part) manipulated as within-subjects and phantom vs. animal as a between-subjects variable. *P* < 0.05 was considered statistically significant. All statistical analysis was performed using the SPSS 20.0.0 software (IBM Corp., Armonk, NY).

## Results

**Figure 2:**
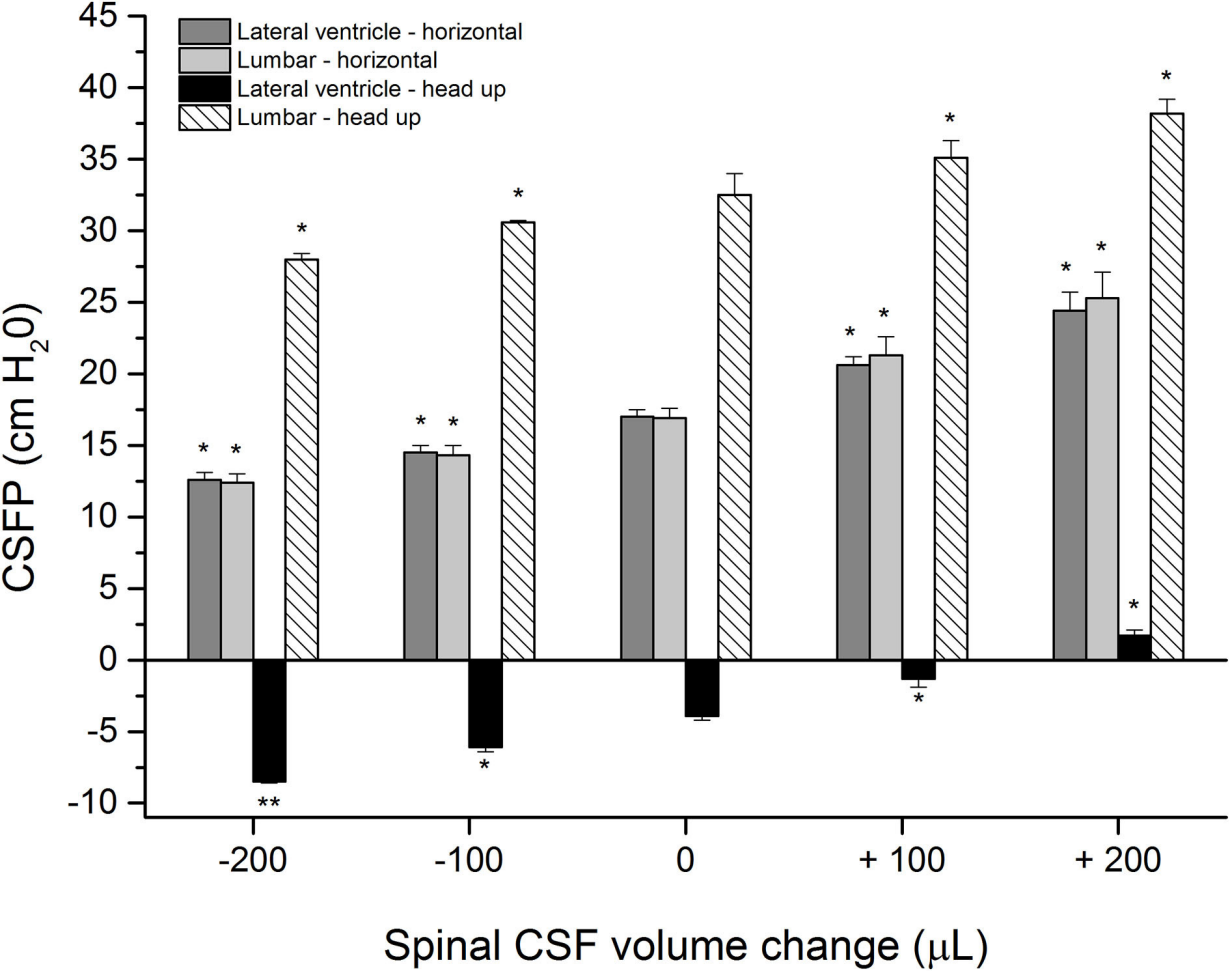
CSFP changes (cm H_2_O) in anesthetized cats in horizontal-0° (lateral ventricle-horizontal and lumbar-horizontal) and head-up 90° (vertical) body position inside the lateral ventricle (lateral ventricle-head-up) and in the lumbar subarachnoid space at L3 level (lumbar-head-up) in control condition (0 μL; *n* = 4), after addition (+) of 100 and 200 μL of artificial CSF into the lumbar space (*n* = 4) and after extraction (-) of 100 or 200 μL from the lumbar space (*n* = 4). Columns represent mean measurement values, while vertical lines represent standard errors of the mean values. There is a statistically significant difference between CSF pressure in the control condition and after adding/removal of fluid volume, *p < 0.05;**p < 0.001.

Control measurements with an animal in a horizontal position show CSFP of 17.0 ± 0.5 cm H_2_O inside the LV and 16.9 ± 0.7 cm H_2_O inside the LSS, while in the head-up position, the pressure was−3.9 ± 0.3 cm H_2_O inside the LV and 32.5 ± 1.5 cm H_2_O in the LSS (*n* = 4) (Figure 2). After the addition of 100 or 200 μL of artificial CSF into the LSS, the CSFP values in the horizontal position increase proportionally inside the LV and LSS (LV = 20.6 ± 0.6 cm H_2_O; LSS = 21.3 ± 1.3 cm H_2_O or LV = 24.4 ± 1.3 cm H_2_O; LSS = 25.3 ± 1.8 cm H_2_O, respectively). Changing of position from horizontal to head-up (vertical) after the addition of 100 μl or 200 μl of a CSF in LSS resulted in a CSFP increase in both LV and LSS (LV = −1.3 ± 0.6 cm H_2_O; LSS = 35.1 ± 1.2 cm H_2_O or LV = 1.7 ± 0.4 cm H_2_O; LSS = 38.2 ± 1.0 cm H_2_O, respectively) (Figure 2).

After placing the animals back into the horizontal position until the CSFP returns to control values, volumes of 100 or 200 μL of CSF were extracted from the LSS, which led to a proportional pressure decrease in LV and LSS both in the horizontal and head-up positions (LV = 14.5 ± 0.5 cm H_2_O; LSS = 14.3 ± 0.7 cm H_2_O or LV = 12.6 ± 0.5 cm H_2_O; LSS = 12.4 ± 0.6 cm H_2_O in horizontal position, LV = −6.1 ± 0.3 cm H_2_O; LSS = 30.6 ± 0.1 cm H_2_O or LV = −8.5 ± 0.1 cm H_2_O; LSS = 28.0 ± 0.4 cm H_2_O in head-up position). Statistically, a significant difference exists between measurements after adding/removal of CSF volume and control values (see Figure 2), which would indicate that CSF pressure both in the cranial and spinal space depends on CSF volume changes in the spinal part.

**Figure 3:**
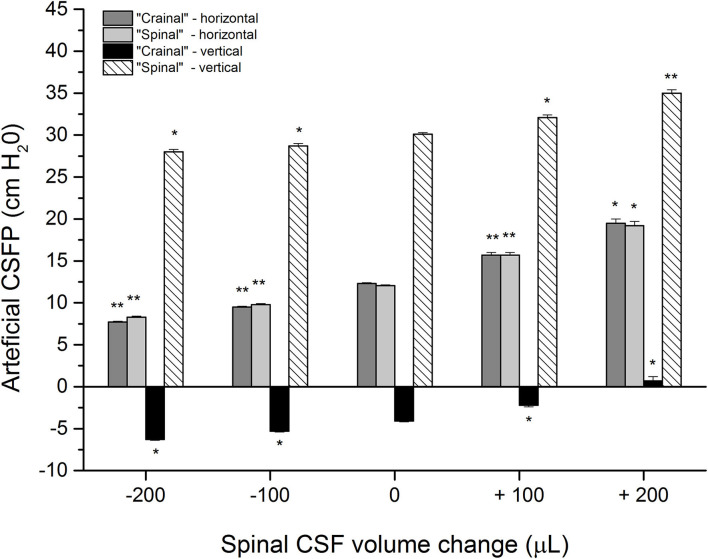
Artificial CSFP changes (cm H_2_O) in the phantom in different positions: horizontal−0° (“cranial”-horizontal; “spinal”-horizontal) and vertical-90°, inside the “cranial” and the “spinal” part of the phantom in the control condition (0 μL; n=5), after addition (+) of 100 and 200 μL of artificial CSF into the “spinal” part (n=5), and extraction (-) of 100 of 200 μL from the “spinal” part (*n* = 5). Columns represent the mean values of the measurements, and vertical lines represent standard errors of the mean values. There is a statistically significant difference between artificial CSF pressure in the control condition and after adding/removal of artificial CSF volume, *p < 0.05;**p < 0.001.

Figure 3 shows the results of corresponding measurements on a phantom with control pressure values in the horizontal and upright position amounting to 12.3 ± 0.1 cm H_2_O and−4.1 ± 0.1 cm H_2_O inside the “cranial” part, while they were 12.4 ± 0.1 cm H_2_O and 30.1 ± 0.2 cm H_2_O in the “spinal” part (five measurements). After the addition of 100 or 200 μL of artificial CSF to the “spinal” part of the phantom, the pressures gradually increase in both parts of the phantom in both positions. The pressure inside the “cranial” part was 15.7 ± 0.3 cm H_2_O and 19.5 ± 0.5 cm H_2_O in the horizontal position, while it was−2.2 ± 0.2 cm H_2_O and 0.7 ± 0.5 cm H_2_O in the upright position. The pressure inside the “spinal” part was 15.7 ± 0.3 cm H_2_O and then 19.2 ± 0.5 cm H_2_O in the horizontal position, while it was 32.1 ± 0.3 cm H_2_O and 35.0 ± 0.4 cm H_2_O in the upright position.

The phantom is then put back to the horizontal position, and 200 μL of artificial CSF is extracted, which allows the pressure to return to control values. This extraction is followed by the removal of 100 or 200 μL of artificial CSF from the “spinal” part of the phantom, which leads to a corresponding decrease of pressure values inside both parts of the phantom, both in the horizontal and upright positions (the pressure inside the “cranial” part was 9.5 ± 0.1 cm H_2_O and 7.7 ± 0.1 cm H_2_O in the horizontal position, while it was−5,3 ± 0,1 cm H_2_O and−6.3 ± 0.1 cm H_2_O in the upright position; the pressure inside the “spinal” part was 9.8 ± 0.1 cm H_2_O and 8.3 ± 0.1 cm H_2_O in the horizontal position, while it was 28.7 ± 0.3 cm H_2_O and 28.0 ± 0.3 cm H_2_O in the upright position). A statistically significant difference can be observed between measurements after adding/removal of artificial CSF volume and control values, which would imply that the artificial CSF pressure inside the system depends on volume changes inside the “spinal” part of the phantom, as was also observed in animals (Figure 2).

In a horizontal position, during the removal of 100 or 200 μL, compliance values vary from 0.039 to 0.045 mL/cm H_2_O in cats and from 0.036 to 0.049 mL/ cm H_2_O in a phantom. On the other hand, after adding 100 or 200 μL of artificial CSF, there is an increase in the CSFP accompanied by a decrease in compliance values. In that case, compliance varied from 0.023 to 0.027 mL/cm H_2_O in cats while the values varied from 0.028 to 0.030 mL/cm H_2_O in a phantom.

There was a slight statistical difference in compliance values between the cats and the phantom in the vertical position, unlike the horizontal one. After verticalization, a significant hydrostatic pressure gradient forms between the measuring points inside the cranial and the spinal space, unlike the horizontal position in which the gradient is practically non-existent. Thus, the compliance values in the cranial and the spinal part of the cat system during the mentioned volumes extraction vary from 0.044 to 0.052 mL/cm H_2_O, and during the addition of the corresponding volumes, they vary from 0.035 to 0.038 mL/ cm H_2_O. After volume removal from a phantom in a vertical position, compliance varied from 0.071 to 0.095, and after volume addition, it varied from 0.035 to 0.052 mL/ cm H_2_O.

## Discussion

### Physiological values of CSFP within CSF system in horizontal and vertical positions

This study indicates that the increase or decrease in CSFP in the cranium can occur due to small changes in the CSF volume in the spinal part without significant changes in the CSF volume in the cranial part of the CSF system. It is further shown that these changes in intracranial CSFP when changing the body's position from horizontal to upright do not depend on the redistribution of fluid volume from the cranium to the hydrostatically lower parts. In this article (Figures 2, 3) and our previous publications (Klarica et al., 2006, 2014), it was shown in control cats that CSFP in the horizontal position is equal in the cranial and spinal parts of the CSF system. However, CSFP inside the cranium (LV) of the control animal in the vertical position is negative (subatmospheric) (Figure 2), and “zero” CSFP is positioned in the foramen magnum region, while inside the LSS, it is highly positive. The observed huge hydrostatic gradient along the CSF system is stable and permanent for an extended period of time (Klarica et al., 2006, 2014). The observed changes in the CSFP in the horizontal and vertical positions of the phantom (Figure 3), in which there is no visible change in the fluid volume in the “cranial” part, do not differ statistically from those observed in cats.

Since CSF forms a freely communicating fluid column inside the craniospinal space with a huge hydrostatic pressure gradient, a question arises about how to explain the appearance of a stable subatmospheric intracranial CSFP in the head-up position. Previously, this occurrence was explained as a transient phenomenon, since the explanation was sought in light of the classical concept of CSF secretion, unidirectional circulation, and absorption (mainly *via* dural venous sinuses), and the changes in the hydrostatic venous blood column Magnaes, 1976a,b; Davson et al., 1987; Valdueza et al., 2000; Gisoff et al., 2004; Qvarlander et al., 2013; Farahmand et al., 2015; Lawley et al., 2015; Holmlund et al., 2018; Linden et al., 2018). Our experiments were performed on a phantom with no CSF secretion, circulation, or absorption and no influence of venous blood hydrostatic pressure. In addition, it is known that the venous blood column in animals and humans is physically separated from the CSF system. Therefore, obtained results on phantom strongly suggest that the combination of the CSF secretion, circulation, absorption, and venous blood changes do not have a crucial influence on acute CSFP changes during the changes of body position or during the addition and removal of a certain fluid volume from the craniospinal space.

**Figure 4:**
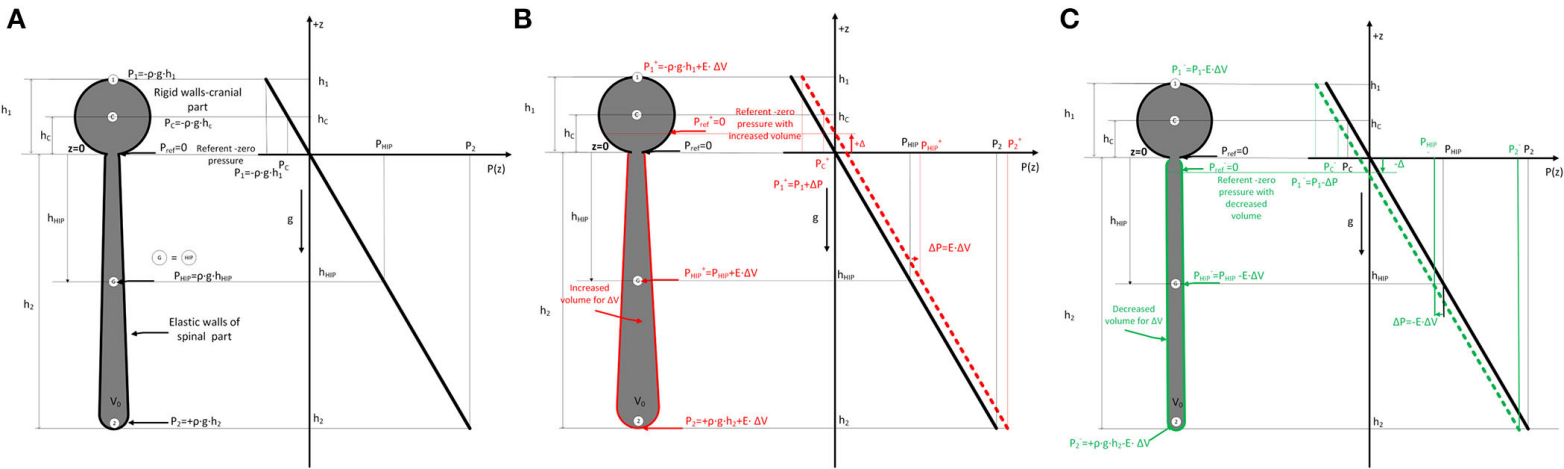
**(A)**. Scheme of the cerebrospinal fluid system in cats (left) consisting of two parts (c-cranial with rigid walls and constant volume, bold black line; spinal part with elastic walls and variable volume) which are filled with fluid. The dimensions of the cranial (h_1_) and spinal (h_2_) parts, as well as the hydrostatic pressure equations, are shown according to the law of fluid mechanics (P = *ρ* x g x h), where ρ is the fluid density, g is the gravitational force, and h is the distance from the opening of the cranium where the pressure is measured. (P_ref_ - reference pressure or zero hydrostatic pressure equal to atmospheric pressure; P_1_ - hydrostatic pressure at the highest point in the cranial part; P_2_ - hydrostatic pressure in the spinal part; P_HIP_ – hydrostatic indifferent point defined by Magnes. Change of fluid pressure (ΔP) in the system with effective elasticity (E) of the spinal part and change of volume (ΔV) is described by equation ΔP=E·ΔV. Therefore, when adding volume **(B)** P= ρ x g x h + E·ΔV (red), and when removing **(C)** P= ρ x g x h - E·ΔV (green). According to the law of fluid mechanics, pressure is increased or decreased for this ΔP value everywhere in the system with shown spatial pressure distribution in the z-direction (right part) with referent fluid volume and increased/decreased fluid volumes. ΔP curve describing the spatial pressure dependence is translated for some value ΔP in all considered points in the system (1,C, G=HIP, 2). The position of the referent point (P_ref_) where the pressure is zero is moved cranially (red arrow) **(B)** or caudally (green arrow) **(C)** (+Δ and -Δ) when a small volume of fluid is added or removed from the system.

All of the observed results from animals and phantom can be accurately explained using the law of fluid mechanics, i.e., differently for the cranial and spinal space. Since in a healthy organism, there is no interruption of fluid continuity within the CSF system, then in accordance with the law of fluid mechanics, hydrostatic pressure can be calculated anywhere within the system (as shown in Figure 4) if the distance from the reference point is known (P = ρ x g x h, where P is the pressure, ρ is the fluid density, g is the gravitational force, and h is the height of fluid column). According to the law of fluid mechanics, inside a space surrounded by rigid walls and an opening at the bottom (like a cranium or a cylindrical “cranial” part of a phantom) (Figure 1), negative pressure appears without changes in fluid volume inside the cranial part (Klarica et al., 2014). Thus, the negative value of the hydrostatic CSFP inside the cranium does not depend on the shape of the volume but only on the distance (h) between the point of measurement and the foramen magnum (P_1_ = - ρ x g x h_1_) (Figure 4). Since the distance of the CSFP (h) measuring cannula in the cranial part of the phantom, as well as in the lateral chamber of cats, was about 4 cm (see Material and Methods section) from the opening of the cranial part of the phantom or foramen magnum, the expected CSFP value in LV and the cranial part of the phantom is−4 cm H_2_O. This value was generally measured at these locations (Figures 2, 3), entirely in line with the law of fluid mechanics. There was also no difference between expected and measured pressures in the spinal part of the phantom, as well as in the LSS of cats (Figures 2, 3), where measuring cannulas were placed 30 and 32 cm away from the opening of the “cranial” part of the phantom and the cat's magna cistern. However, according to the law of fluid mechanics, the pressure values were positive in the spinal part (P_2_ = *ρ* x g x h_2_) (Figure 4).

If the cerebrospinal fluid pressure behaves in accordance with the law of fluid mechanics, it is to be expected that in the upright position, the pressure in the cranium will be lower than the atmospheric pressure all the time while the body is in the upright position. In our previous studies on cats, we observed that CSFP in the cranium was stable and negative for 75 min while the animal was in the head-up position (Klarica et al., 2014). Clinical studies of CSFP in cranium using telemetric methods in healthy and ill subjects also show such a phenomenon in humans. When changing the position of the body from supine to head-up position (sitting or standing), CSFP in the cranium always falls and takes a long-lasting negative (subatmospheric) value (Andresen et al., 2015; Ertl et al., 2017; Rot et al., 2020).

In contrast to previous observations in the open cranium (Davson et al., 1987) that verticalization leads to a collapse of veins within the cranium, new research on patients with closed cranium using CT has shown that verticalization of patients causes a prominent reduction (collapse) in the volume of neck vein vessels but with almost no change in the volume of intracranial vessels (Kosugi et al., 2020). It seems that described intracranial blood behavior, unlike CSF, has no significant effect on pressure regulation during verticalization. The observed results from animals and humans indicate that subatmospheric CSFP in the cranium in the upright position can be a long-lasting phenomenon, which is the consequence of the influence of gravity predominantly on the CSF system.

### CSF micro-volume changes in spinal space affect CSFP in different positions

To explain the changes in CSFP in the head-up position when adding or extracting the volume of artificial CSF from spinal space and moving the zero point of CSFP cranially or caudally, we introduced a simple linear model in accordance with the law of fluid mechanics shown in Figure 4. By using a simplified model of the CSF system (see Figure 4), it is observed that hydrostatic pressure (ΔP) anywhere inside the CSF system is increased/decreased when the mass (Δm)/volume (ΔV) of CSF is added/removed into/from the system. That simplified linear model connects the change of internal pressure (ΔP) in the system with effective elasticity (E) of the spinal part and change of volume (ΔV) by using equation (1) (Landau et al., 2009):


(1) $$\begin{array}{r} \text{Δ }P\;=\;E\;•\text{Δ }V \end{array}$$

For the cranial part with rigid walls, the elasticity is zero, so the effective elastance (E) is defined with only the elastance of the spinal part (E) because the system can be observed as two coupled systems (rigid+elastic) connected in parallel. If we add or remove a small amount of fluid volume (ΔV), the pressure inside (P) is changed due to increased or decreased tension of spinal walls, and all points in the system are at a higher or lower level (see Figure 4).

The pressure increase, for example, in the reference point G (and at any point in the system) and all other considered points is given with Eqs 2a (fluid added) and 2b (fluid removed) with known effective elastance of spinal part (E) of the system.


(2) $$\begin{array}{l} P_{G}^{+}=P_{G}+E•\Delta V \end{array}$$

(3) $$\begin{array}{l} P_{G}^{-}=P_{G}-E•\Delta V \end{array}$$

The pressure (P) is increased or decreased for this ΔP value everywhere in the system, as shown in Figure 4, according to the hydrostatic pressure law with shown spatial pressure distribution in the z-direction with referent fluid volume and increased/decreased fluid volumes. In the first approximation, the dimension change in the elastic part is negligible in the axial direction, and there is a small increase in radial directions, so there is no change in the z-direction. Due to the change of pressure ΔP, the curve describing the spatial pressure dependence is translated for some value of ΔP at all points in the system. Also, the position of the referent point (P_ref_) where the pressure is zero is changed to up (red) or down (green) for differential height (+Δ and -Δ) when a small volume of fluid is added or removed from the system. This is shown for all considered points (1,C,G=HIP, 2), and increased or decreased values of the pressure are shown with superscript + or -.

The measured CSFP changes in cats and phantoms are consistent with the previously presented theoretical analysis and equations for calculating CSFP changes when adding/removing fluid volume in the spinal space (see Supplementary material). It is important to emphasize that in our research, CSFP changes inside the cat or phantom cranial and spinal space during the addition and extraction of 100 or 200 μL of fluid do not differ significantly and that the hydrostatic gradient between the measuring points in the cranium and the spinal part was constant. For example, when we take off a certain CSF volume from the LSS in cats, both lumbar and cranial CSFP decreased, and zero CSFP point descended for approximately 2.0–2.5 cm for each extracted 100 μL (Figures 2, 4). On the other hand, adding the same CSF volume causes both lumbar and cranial CSFP to increase, and the zero CSFP point moves cranially for 2.6–3.0 cm.

The experiments on a phantom (Figure 3) showed that the addition of 200 μL leads to a pressure increase inside the “cranial” and “spinal” parts and moves the zero pressure value for about 4.8 cm cranially. Conversely, extraction of 200 μL leads to a pressure decrease in the “cranial” and “spinal” parts, which implies that zero pressure point moved from the opening of the “cranial” part of the phantom for 2.2 cm caudally. Thus, the experiments on a phantom and animals support our hypothesis that the observed variability of the zero CSFP value in vertical body position could depend on the fullness of the CSF system in its spinal part.

The ability of craniospinal space to adjust to the volume changes is usually described as spatial compensation (translocation of CSF from the cranial to the spinal space and increase of CSF absorption according to classical hypothesis) and craniospinal compliance (reflects acute volume–pressure relationship, Δ volume/Δ pressure) (Davson et al., 1987; Portella et al., 2005; Lawley et al., 2015; Ocamoto et al., 2021; Löfgren and Zwetnow, 1973). Craniospinal compliance and spatial capacity determine the slope of the volume–pressure curve. There is an exponential relationship between the CSFP changes and craniospinal compliance. According to the well-known phenomena, the value of compliance decreases with CSFP increase, which can also be observed in our research on cats. That is, in those experiments, compliance calculated during the volume removal and pressure decrease (about 0.042 mL/cm H_2_O) was bigger than the compliance calculated in the case of volume addition and subsequent pressure increase (about 0.025 mL/ cm H_2_O). Since the addition or removal of a certain fluid volume from the spinal CSF space leads to the same pressure changes both cranially and spinally, together with preserved hydrostatic gradient, it is clear that during those pressure changes, the same compliance value will be obtained both inside the cranium and inside the spinal space. Thus, pressure changes inside the cranium enable us to calculate only the compliance of total craniospinal space (Ocamoto et al., 2021). This is generally true for all the experiments done on animals and humans if there is normal communication between the cranial and the spinal space. It is generally accepted that CSF physiology is not different between animal species and humans.

A slight difference in obtained compliance values in an upright position between the model and the animals, especially in the case of volume extraction, could be due to a somewhat better elasticity of the spinal part of the phantom, which is made of rubber compared to the spinal dura mater. Very small differences in pressure could also be the consequences of the certain possibilities of spatial adjustment of brain and blood (e.g., vasoconstriction and vasodilatation) volume within the animal cranium. However, the results of our study done on cats and phantoms in which there is no intracranial compliance, nor the possibility of spatial compensation, suggest that the spinal space plays a crucial role in the regulation of the intracranial CSFP.

### Implications in neurological disease

According to our new concept, the cranium seems to play a significant role in body uprightness and position changes (important from an evolutionary point of view) because it prevents sudden changes in the volume of intracranial fluids. Thus, in a study on patients with idiopathic intracranial hypertension (IIH) using automated MRI measurements of the craniospinal CSF volume, it was observed that after the lumbar CSF withdrawal drop in intracranial pressure is primarily related to the increase in spinal compliance and not cranial compliance due to the reduced spinal CSF volume and the nearly unchanged cranial CSF volume (Alperin et al., 2016). Furthermore, the same phenomenon (preservation of intracranial cerebrospinal fluid volume with large changes in the spinal cerebrospinal fluid volume) was observed in a patient with IIH whose LP shunt was replaced by a VP shunt due to severe headaches and over drainage (Nikić et al., 2016). These clinical observations in which significant changes in the spinal cerebrospinal fluid volume occur without significant cranial CSF volume changes strongly support our understanding of intracranial fluid behavior and CSFP regulation, as shown in Figures 2, 3.

In human physiological conditions, such experimentally induced changes of the spinal CSF volume could happen due to abdominal straining during defecation, lifting weight, sneezing, crying of a baby, etc., which can cause pressure to increase inside the abdominal and thoracic veins, and subsequently increase of the CSFP (Davson et al., 1987). Therefore, this could lead to a disruption of blood flow in epidural venous plexuses, an increase in blood volume inside the epidural space, and additional force which influences CSF volume inside the lumbar part of the system, which, as we have shown (Figures 2, 3), should lead to an increase of the CSFP inside the cranium and consequently cause a shift of the zero pressure position.

Our proposed hypothesis and results shown in Figures 2, 3 could help us understand why patients with spinal liquorrhea have worse clinical status in upright positions than those with cranial liquorrhea (Levine and Rapalino, 2001). According to our hypothesis, in the case of cranial liquorrhea, during body verticalization, the cerebrospinal fluid pressure inside the cranium reaches even subatmospheric values, which stops liquorrhea from the cranium. However, such patients poorly tolerate supine positions because the cerebrospinal fluid pressure in the cranium becomes positive. In the case of spinal liquorrhea, CSF leakage during supine position is less pronounced than in upright position. In an upright position, CSF pressure becomes more positive inside the spinal subarachnoid space. Our proposed hypothesis could explain why lumboperiotneal shunting has common complications, such as over drainage, intracranial hypotension, and postural headache.

It seems that negative intracranial CSFP in the upright position is a long-lasting phenomenon (Klarica et al., 2014). This indicates that cerebral perfusion pressure should be significantly higher than previously believed during two-thirds of the day while people are in the upright or sitting position. According to our concept, it seems that in people in an upright position, the blood supply to the brain is much better, and thus evolutionarily adapted for verticalization and bipedal walking.

Since cranial intradural volume cannot change significantly, unlike the spinal one (Davson et al., 1987), it is to be expected that a tendency to decrease CSF volume inside the cranium (e.g., extraction of cerebrospinal fluid from LV or cortical subarachnoid space), that volume has to be compensated instantly, because the vacuum inside the space, such as the cranial cavity, cannot be created. Therefore, it is most likely that redistribution of CSF volume from the spinal into the cranial space occurs (Orešković et al., 2018). It is necessary to stress that this shift of CSF volume inside the CSF system finally leads to a decrease in the CSF volume inside the spinal part with an instant decrease of CSFP in the cranium (explained by our new concept, Figure 4). The described phenomenon may explain how the reduction of CSFP occurs when applying iv. hyperosmolar mannitol solutions (Orešković et al., 2018), because in the first 30 min after application, there is a decrease in CSFP without changes in the percentage of water in the brain, cerebral blood volume, or intracanal fluid. As mortality in humans caused by high CSFP is a serious/major health problem, new insights into the regulation of CSFP should be very important in preventing and treating this pathological condition.

The presented results strongly support our hypothesis of CSF physiology where CSF/interstitial fluid volume depends on osmotic and hydrostatic force gradient between capillaries and interstitial space inside cranial and spinal CNS tissues (Orešković and Klarica, 2010; Orešković and Klarica, 2011; Bulat and Klarica, 2011; Orešković et al., 2017a,b; Klarica et al., 2019; Radoš et al., 2021). Until today, the regulation of CSFP was explained by the classic hypothesis of CSF physiology, e.g., secretion, circulation, and absorption of CSF. A famous equation that links the classical concept of CSF physiology and intracranial CSFP is CSFP = Vf x Ro + Pv, which suggests that intracranial CSFP has to be positive because the CSF secretes, flows unidirectionally, and absorbs all the time.

Since under the same experimental conditions, changes in CSFP obtained in phantom (Figure 3) reflect the results of CSFP obtained in cats (Figure 2), it is obvious that the classical hypothesis cannot explain CSFP regulation and long-lasting negative CSFP. Therefore, there is neither secretion, circulation, nor absorption in the phantom, and the regulation of “CSFP” could be explained only by the law of fluid mechanics. Thus, “CSFP” behavior in the phantom, compared with its behavior in anesthetized cats, offers us the opportunity to propose that the law of fluid mechanics is a unique explanation of CSFP regulation in different body positions. The use of phantom as a model that faithfully reflects the craniospinal system of cats is therefore of vital importance because the comparison of experimental results allows us to gain new insights into the behavior of CSF volume in the CSF space.

The results of this work, as well as our previous research (Klarica et al., 2006, 2014, 2019; Orešković et al., 2018), suggest that the role of spinal subarachnoid space in the regulation of CSFP is crucial and is consistent with research results that give a significantly greater role in compensation of intracranial hypertension by spinal rather than intracranial space (Löfgren and Zwetnow, 1973; Magnæs, 1989; Alperin et al., 2021). In addition, the existence of craniospinal communication within the cerebrospinal fluid space appears to be important not only to compensate for the increase in cranial pressure but also to enable therapeutic reduction of CSFP within the entire CSF system. That is, the obstruction of craniospinal communication would prevent the reduction of CSFP in the cranium, which, according to our results, would occur due to the reduction of the volume in the spinal space.

## Limitations

There are some limitations to this study. In cats, cerebrospinal fluid volumes in the cranial and spinal parts of the CSF system were not determined by adequate volumetric methods. We expect that the combination of neuroradiological research with the help of MR, which allows recording in the head-up position, and the method of long-term measurement of CSFP in various diseases and body positions will confirm our results in the future. This study measured pressure changes in the cranial and spinal spaces that had normal communication. Therefore, from the obtained results, it is impossible to precisely determine the value of compliance and elastance separately for the cranial and spinal spaces.

## Conclusion

The matching results of the CSFP measurements obtained under the same experimental conditions in cat and phantom suggest that the spinal space contributing to the compliance of the total craniospinal space is significantly more than the cranial space, since the results obtained on a phantom without the possibility of the cranial compliance existence can approximate the system very well. The change of the micro-CSF volume in the spinal CSF region also causes a significant CSFP change inside the cranium, i.e., even a minor spinal volume change substantially changes the cranial CSFP. The observed clinical phenomenon of moving the zero (atmospheric) point of the CSFP in the human vertical position could be explained mainly by changes in the CSF volume in the spinal part of the CSF system and a simultaneous increase or decrease of the added volume (CSF, blood, tissue edema, etc.) in the spinal area. The obtained phenomenon is possible to explain only in accordance with the new hypothesis of CSF physiology.

## Data availability statement

The raw data supporting the conclusions of this article will be made available by the authors, without undue reservation.

## Ethics statement

The animal study was reviewed and approved by Ethics Committee of the University of Zagreb Medical School. Written informed consent for participation was not obtained from the owners because the owners were verbally informed about the experimental protocol, which was previously approved by the official Ethical Committee.

## Author contributions

MK and DO designed and conceptualized the study. MK, MR, GE, and IJ performed experiments on animals and phantom. AP and ZV introduced a theoretical model to explain the results. MR, MK, and DO prepared the figures, drafted the initial version of the manuscript, and explained the presented results according to their new hypothesis of CSF physiology. All authors have read and approved the final draft of the manuscript.

## Funding

This work has been supported by the Croatian Science Foundation and the Ministry of Science and Education of the Republic of Croatia (Projects: 1. Pathophysiology of cerebrospinal fluid and intracranial pressure: No. 108-1080231-0023; and 2. Serotonergic modulation of obesity: cross-talk between regulatory molecules and pathways: No. IP-2014-09-7827). The research was co-financed by the Scientific Centre of Excellence for Basic, Clinical, and Translational Neuroscience (project Experimental and clinical research of hypoxic-ischemic damage in perinatal and adult brain; GA KK01.1.1.01.0007 funded by the European Union through Europe).

## Conflict of interest

The authors declare that the research was conducted in the absence of any commercial or financial relationships that could be construed as a potential conflict of interest.

## Publisher's note

All claims expressed in this article are solely those of the authors and do not necessarily represent those of their affiliated organizations, or those of the publisher, the editors and the reviewers. Any product that may be evaluated in this article, or claim that may be made by its manufacturer, is not guaranteed or endorsed by the publisher.
