# Supplementary material for: Cerebrospinal fluid micro-volume changes inside the spinal space affect intracranial pressure in different body positions of animals and phantom
Source: Front Mol Neurosci. 2022 Sep 14;15:931091. doi: 10.3389/fnmol.2022.931091 (PMC9518230; doi:10.3389/fnmol.2022.931091)

SUPPLEMENT MATERIAL: Cerebrospinal fluid micro-volume changes inside the spinal space affect intracranial pressure in different body positions of animals and phantom

Mathematical model

Figure S1 schematically shows the measuring points (C and 2) and hydrostatic indifferent point (G). In a static fluid, the pressure rises in the direction of the gravity vector ($\vec{g}$). In the vertical (head up) position $(\theta = 90°)$ the pressure difference in the two measuring points is $P_2 - P_C = \Delta P_{vertical} = \rho \cdot g \cdot h_{2Cv}$ and in the horizontal position, the pressure difference is $P_2 - P_C = \Delta P_{horizontal} = \rho \cdot g \cdot h_{2Ch}$. The angle θ in horizontal position is defined as $\theta_h = \tan^{-1}(\Delta P_{horizontal} / \Delta P_{vertical})$. $\Delta P_{vertical}$ and $\Delta P_{horizontal}$ are defined as the arithmetic mean from the measurements in head up and horizontal position, respectively. The pressure difference in points 2 and G in vertical position is defined as $P_2 - P_G = \Delta P_{2G} = \rho \cdot g \cdot h_{2G}$, where h_{2G} is unknown.

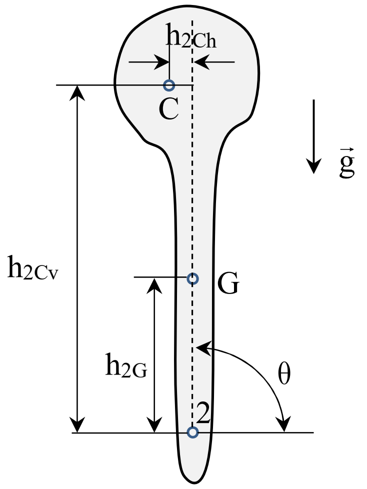

Figure S1. Position of measuring points (C = in cranial part, 2 = in spinal part) and hydrostatic indifferent point (G)

With the assumption of the rigid cranial part and elastic spinal part (with constant compliance $C = 1/E = \Delta V / \Delta P_G$, where E is the elastance), the mathematical model reads

$$P_C = P_2 - \Delta P_{vertical} \cdot \sin(\theta), \quad (1)$$

$$P_2 = P_G + E \cdot \Delta V + \Delta P_{2G} \cdot \sin(\theta), \quad (2)$$

where ΔV is the spinal CSF volume change. The free model parameters are P_G, E, and ΔP_{2G}. Model parameters were identified from pressure measurements of P_C and P_2 in horizontal $(\theta=\theta_h)$ and vertical position $(\theta=90°)$ for ΔV = -0.2; -0.1; 0, 0.1 and 0.2 ml (total 10 measurements) by using the least square method in which the following RMS (Root Mean Square) was minimized:

$$RMS=\sqrt{\frac{\sum_{i=1}^{10}\left(P_{Ci}^{measured}-P_{Ci}\right)^2+\sum_{i=1}^{10}\left(P_{2i}^{measured}-P_{2i}\right)^2}{20}}$$

The method was implemented in GNU Octave (https://www.gnu.org/software/octave/index) free software, and minimization was performed by using the function *fminsearch*.

Results for four cats

Parameter	Value	Unit
$\Delta P_{vertical}=$	34.4	cmH_2O
$\Delta P_{horizontal}=$	1.1	cmH_2O
$P_G=$	20.4	cmH_2O
E=	31.4	cmH_2O/ml
C=	0.0318	ml/ cmH_2O
$\Delta P_{2G}=$	10.1	cmH_2O
RMS=	1.64	cmH_2O

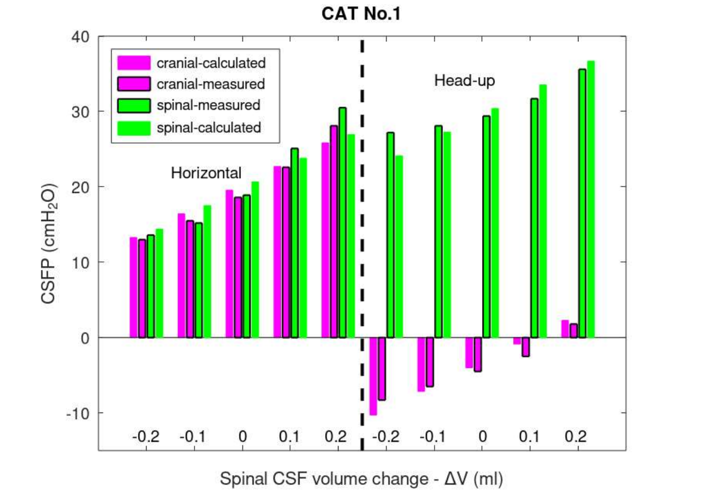

Parameter	Value	Unit
$\Delta P_{vertical}=$	36.1	cmH_2O
$\Delta P_{horizontal}=$	-0.82	cmH_2O
$P_G=$	16.3	cmH_2O
E=	26.0	cmH_2O/ml
C=	0.0385	ml/ cmH_2O
$\Delta P_{2G}=$	16.0	cmH_2O
RMS=	0.59	cmH_2O

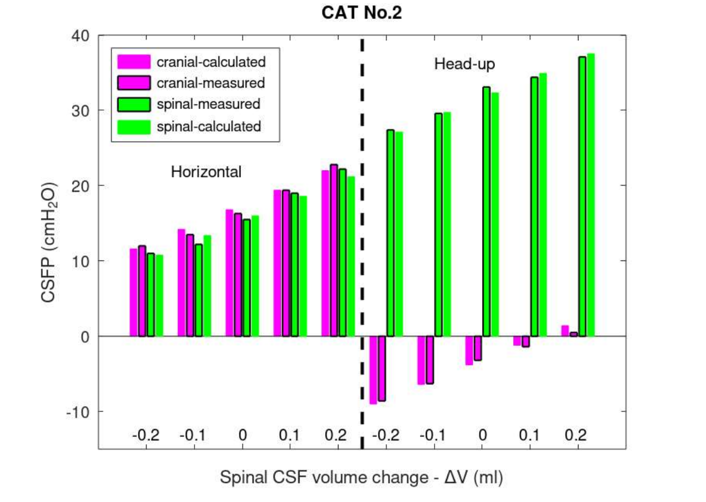

Parameter	Value	Unit
$\Delta P_{vertical}=$	37.2	cmH_2O
$\Delta P_{horizontal}=$	0.14	cmH_2O
$P_G=$	17.2	cmH_2O
E=	28.8	cmH_2O/ml
C=	0.0346	ml/ cmH_2O
$\Delta P_{2G}=$	17.1	cmH_2O
RMS=	0.53	cmH_2O

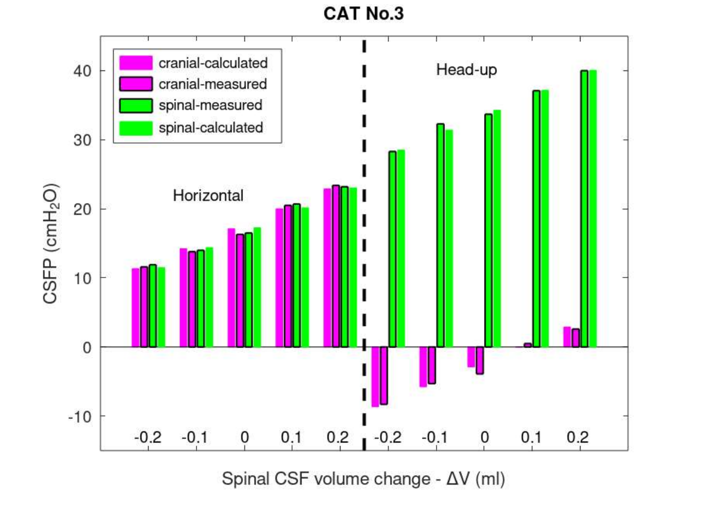

Parameter	Value	Unit
$\Delta P_{vertical}=$	38.3	cmH_2O
$\Delta P_{horizontal}=$	0.46	cmH_2O
$P_G=$	18.1	cmH_2O
E=	26.3	cmH_2O/ml
C=	0.0380	ml/ cmH_2O
$\Delta P_{2G}=$	16.5	cmH_2O
RMS=	0.72	cmH_2O

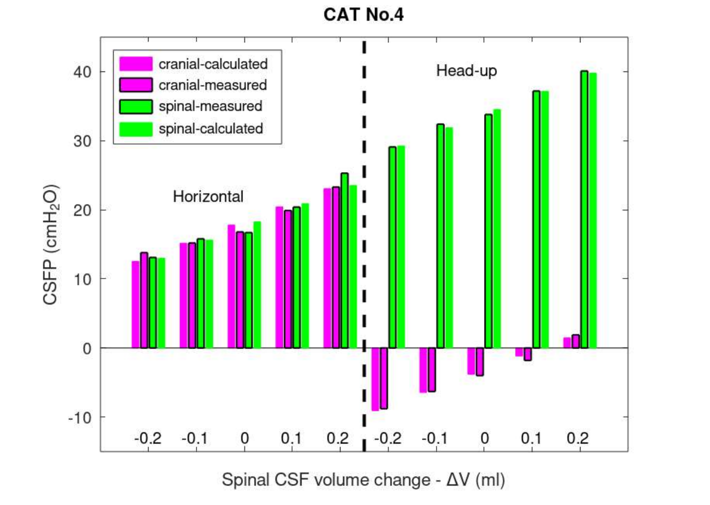

Supplement: Supplementary file 1 [file Data_Sheet_1.PDF]
